# Interventions to prevent and treat multiple long-term conditions and their consequences across the life course: concepts and definitions

**DOI:** 10.1186/s12916-026-04838-4

**Published:** 2026-04-10

**Authors:** Miles D. Witham, Guruprasad P. Aithal, Michelle Collinson, Rachel Cooper, Melanie J. Davies, Andrew Farmer, Simon D. S. Fraser, Peter Hanlon, Kamlesh Khunti, Sarah E. Lamb, Claire McDonald, Krishnarajah Nirantharakumar, Avan A. Sayer, Nira Shah, James P. Sheppard, Stephanie J. C. Taylor, Farheen Yameen, Lily Yao, Sally J. Singh, The Multiple Long-Term Conditions Cross-NIHR Collaboration Interventions and Prevention Workstream

**Affiliations:** 1 NIHR Newcastle Biomedical Research Centre, Newcastle Upon Tyne Hospitals NHS Foundation Trust, Cumbria Northumberland Tyne and Wear NHS Foundation Trust and Newcastle University, Newcastle Upon Tyne, UK; 2 AGE Research Group, Translational and Clinical Research Institute, Faculty of Medical Sciences, Newcastle University, Newcastle Upon Tyne, UK; 3 NIHR Nottingham Biomedical Research Centre, Nottingham, UK; 4 Leeds Clinical Trials Research Institute, University of Leeds, Leeds, UK; 5 Diabetes Research Centre, University of Leicester, Leicester, UK; 6 NIHR Leicester Biomedical Research Centre, Leicester, UK; 7 NIHR Oxford Biomedical Research Centre, Oxford, UK; 8 School of Primary Care, Population Sciences, and Medical Education, University of Southampton, Southampton, UK; 9 School of Health and Wellbeing, University of Glasgow, Glasgow, UK; 10 NIHR Applied Research Collaborative East Midlands, Leicester, UK; 11 NIHR Exeter Biomedical Research Centre, Exeter, UK; 12 Faculty of Health and Life Sciences, University of Exeter, Exeter, UK; 13 Department of Population Health Sciences, King’s College London, London, UK; 14 Multiple Long-Term Conditions Cross-NIHR Collaboration, Newcastle Upon Tyne, UK; 15 Nuffield Department of Primary Care Health Sciences, University of Oxford, Oxford, UK; 16 Wolfson Institute of Population Health. Queen Mary University of London, London, UK; 17 Department of Cardiovascular Sciences, University of Leicester, Leicester, UK; 18 Department of Respiratory Sciences, University of Leicester, Leicester, UK

**Keywords:** Multimorbidity, Multiple long-term conditions, Life course, Interventions, Prevention, Taxonomy

## Abstract

Multiple long-term conditions (MLTC; also known as multimorbidity) constitute an important unmet health challenge. Designing and evaluating interventions to prevent and treat MLTC and their consequences is essential but difficult, due to heterogeneous risk factors, biological mechanisms, condition combinations, social and functional impacts of MLTC. To make progress, shared ways of describing and discussing interventions to prevent and treat MLTC and their consequences would bring consistency and clarity to this complex research area.

In this position paper, we aim to provide a framework to address this recently articulated need. We propose a conceptual framework for placing MLTC interventions on a spectrum of prevention and treatment (from primordial to quaternary prevention). We consider different key intervention time points across the life course and how the focus of interventions may change from reducing risk factors in earlier life, through prevention of condition accumulation or progression in later life, to mitigating adverse consequences on symptoms and function as conditions progress. We propose taxonomies of interventions based on simple description but also on the existing World Health Organisation International Classification of Health Interventions taxonomy. We discuss principles of intervention development, focussing particularly on the need to target shared biological mechanisms and risk factors rather than attempting to treat or prevent each condition individually, and focussing on symptoms or consequences of MLTC that are prioritised by patients.

Finally, we consider the attributes of effective MLTC interventions; we argue that ideal interventions should have robust evidence of efficacy and effectiveness, should be scalable and implementable within existing health and social care systems, should reduce (or at least not increase) health inequality, should be cost-effective, and should deliver major improvements over and above existing interventions, either at individual or population level. We recommend using this conceptual framework, taxonomy and criteria for assessing interventions together to organise the planning and evaluation of MLTC intervention research thereby accelerating progress in this important yet understudied field.

## Background

### Rachel Cooper, Peter Hanlon, Kamlesh Khunti, Avan Sayer, James Sheppard

Multiple long-term conditions (MLTC, also known as multimorbidity [1]; also see Table 1) refer to the coexistence of two or more chronic health conditions affecting an individual [2, 3]. MLTC significantly impact individuals, communities, and healthcare services. People living with MLTC have poorer health-related quality of life and are at greater risk of hospital admission and premature death [4–6]. People living with MLTC often experience greater treatment burden resulting from multiple treatments and appointments focussing on individual conditions, conflicting treatment priorities and fragmented healthcare [7–9]. The presence of MLTC is also associated with greater use of social care, including the need for care home admission [10].

**Table 1:** A note on terminology used in this paper

Multiple long-term conditions (MLTC): The presence of two or more long-term health conditions [3]. These may include physical or mental health conditions, and may include non-infectious or infectious conditions. No one condition is prioritised [11]
Multimorbidity: Alternative term for MLTC. MLTC is the term preferred by people living with MLTC in the UK [1]
Comorbidity: Additional conditions present alongside an index condition which is the main focus [11]

MLTC are common and the prevalence is increasing [12–15]. While the prevalence of MLTC varies considerably dependent on the characteristics of the population being studied and conditions included, it has been estimated that approximately one third of adults worldwide are living with MLTC, and more than half of those with any long-term condition have MLTC [16–19]. This is in part a consequence of the ageing population, with the improvements in healthcare over time leading to longer survival of people living with a wide range of long-term health conditions [20]. A recent population-based study of over 60 million people in England estimated the overall prevalence of MLTC to be 14.8% and this varied with age from 0.9% (in those aged 0–19 years) to 68.2% in those aged 80 years and over [21].

While the scale and impact of MLTC are increasingly apparent, we currently lack robust evidence of how best to intervene to prevent, treat or mitigate the impacts of MLTC [2, 3, 22, 23]. Many existing interventions and services focus on specific diseases, but effectively addressing the challenge of MLTC requires a holistic approach encompassing all long-term conditions. This is distinct from the perspective usually adopted when considering ‘comorbidity’, the presence of one or more additional long-term conditions in the context of an index condition, which is the primary focus of interest [1, 11]. MLTC therefore requires a paradigm shift in how we design, deliver and evaluate interventions.

A legitimate question is the extent to which MLTC require a different framing to the way that we consider single conditions. One answer to this question is that current paradigms for single conditions clearly do not translate sufficiently well to MLTC to deliver outcomes that people living with MLTC find acceptable, and frameworks with a specific focus on MLTC are therefore necessary. Within these conceptual frameworks, there are elements that are likely to be qualitatively different for MLTC (e.g. the need for condition-agnostic outcome measures, potential for conflict between sets of guidance), and other elements that are similar in nature, but different in degree to issues that are important for single conditions (e.g. well-established disease risk factors and protective factors, the higher risk of side-effects and the greater complexity of care).

MLTC are, by their nature, a heterogeneous concept. Different people experience different combinations of conditions with varying degrees of severity, at different life stages and influenced differently according to social determinants of health. Attempts to characterise MLTC range from counts of conditions (weighted or unweighted) [24, 25], identification of ‘complex multimorbidity’ (which has been conceptualised as MLTC in which conditions span several body systems [26]), clustering approaches seeking to identify frequently co-occurring conditions that may share common risk factors and therefore benefit from similar interventions [27], and syndemics focussing on interactions between individual conditions, context and environment [28]. Potential approaches to prevent, treat and mitigate the impacts of MLTC are therefore diverse. The heterogeneity of MLTC combinations, severity, consequences and affected populations thus extends into heterogeneity around how we conceptualise and operationalise interventions to prevent and treat MLTC, potentially leading to a lack of consistency in how interventions are described in terms of their design, targets, delivery and evaluation. Consensus exercises have attempted to improve heterogeneity in the definitions of measurements and hence improve study compatibility and reproducibility [29].

In this position paper, we aim to provide a framework to answer the recently articulated need for a consistent way of discussing interventions to prevent and treat MLTC and their consequences [30]. We propose a conceptual framework for placing MLTC interventions on a spectrum of prevention and treatment with different key intervention time points across the life course; we propose a taxonomy of interventions and we consider the attributes of effective MLTC interventions to facilitate future discussion of issues around design, delivery and evaluation. We anticipate that this framework will be of assistance to researchers seeking to design and evaluate interventions, researchers seeking to synthesise existing literature, but also to policymakers and service delivery teams seeking to select and implement interventions to manage and prevent MLTC.

Declaration of competing interests: None.

## Interventions for prevention and treatment of MLTC—a conceptual framework

### Simon Fraser, Krishnarajah Nirantharakumar, Stephanie Taylor

Traditional concepts of interventions for prevention and treatment, both at individual and population levels, are challenging to apply to MLTC and are not yet well reported or researched [2, 31]. Current models for describing interventions commonly consider the World Health Organisation (WHO) classes of prevention: primordial, primary, secondary, tertiary and quaternary prevention, the latter classes often focussing on individual therapeutic interventions [32]. Although this conceptual framework uses the language of prevention, the distinction between prevention and treatment is somewhat artificial and the secondary, tertiary and quaternary prevention aspects of this framework encompass interventions that are analogous to treatments for conditions or their consequences.

*Primordial prevention* aims to reduce the ‘emergence and establishment of environmental, economic, and social conditions that are known to increase the risk of disease’, typically at a health and social policy level that often includes the wider social determinants of health [33]. *Primary prevention* aims to reduce the risk of a disease occurring and can include population-level or individual-level interventions aimed at improving health through minimising exposure to risk factors, enhancing exposure to health-promoting factors, and reducing the adverse impact of social and economic determinants of health. *Secondary prevention* aims to detect disease and manage it early enough to improve health outcomes and prevent recurrent disease. *Tertiary prevention* involves activities to mitigate the impacts of ill-health and conditions that have ongoing, long-term effects and prevention of collective outcomes including frailty and loss of independence and function including activities of daily living and ability to work [32]. Such a framework can be extended further to include prevention of overmedicalisation and iatrogenic harms (e.g. from polypharmacy)—*quaternary prevention* [34].

**Fig. 1:**
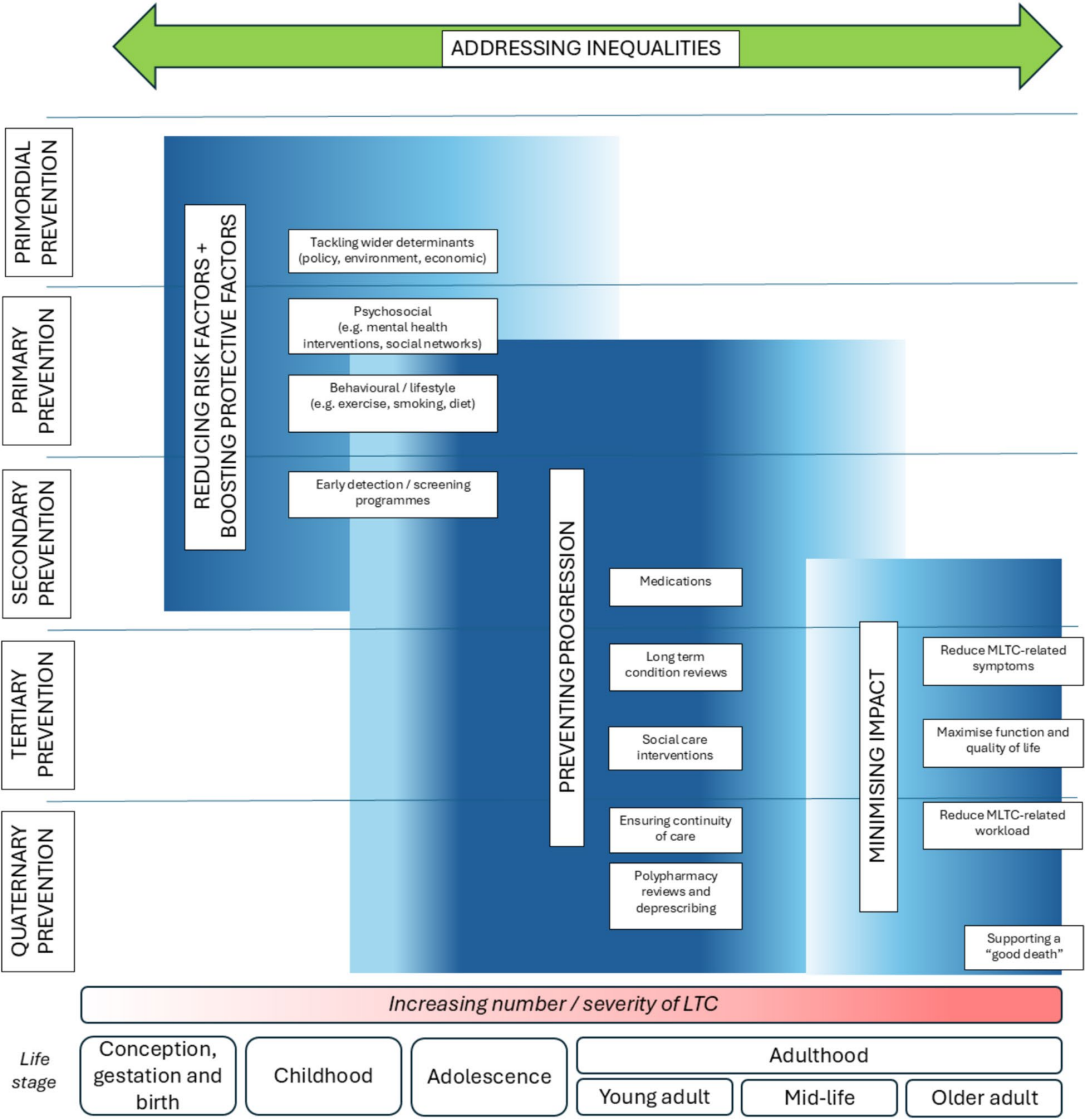
Framework for classes of MLTC interventions Depth of shading indicates relative importance at a particular stage of the life course

There are limitations in applying this terminology to MLTC. Whilst there are common underlying risk factors for some conditions and other broader health determinants that contribute to the development of many long-term conditions (LTC), risk factors, detection strategies and management for individual conditions and individual people can vary widely [2, 31]. We therefore propose a modified framework for defining classes of interventions for MLTC, building on the primordial to quaternary prevention strategy framework. Figure 1 summarises this conceptual framework and shows how the emphasis on different types of intervention may shift across the life course.

#### Reducing risk factors and boosting protective factors

Many LTCs have common modifiable risk factors, such as obesity and smoking, and are often closely associated with health inequalities and wider determinants of health [35, 36]. Strategies aimed at reducing these risk factors and boosting protective factors at both individual and population levels such as those described for primordial and primary prevention are likely to reduce the incidence of MLTC. At a population level this would include policies and societal interventions aimed at addressing the wider determinants of health such as the environment, the local economy, housing, employment and education and those relating to health such as tobacco and alcohol control, physical behaviours (activity, sedentariness and sleep) and diet, but also intersectoral policies that address mental health, substance misuse and domestic violence [32]. At an individual level this could include provision of information about common health risks, with measures to address these within primary care such as through the NHS Health Check programme, as well as smoking cessation, weight management and exercise referral programmes. Many existing prevention initiatives are already set up to address risk factors for several conditions (and thus are likely to be appropriate for prevention of MLTC) but there may be a place for altering the messaging used by such programmes if public awareness of MLTC as an undesirable health state becomes more widespread.

#### Preventing progression

To prevent progression, early detection is important, for example via targeted screening programmes or use of algorithms using routinely-collected clinical data to detect those at high risk. For LTC that have been detected, the aim should be early detection to both prevent worsening severity or development of further conditions. Measures to address this are largely those covered by secondary prevention. Work currently underway to predict the next likely condition in patients with MLTC may in the future play a role in targeting interventions [37]. Preventing progression may include targeted screening for concordant conditions (i.e. conditions with closely related risk factors and pathology, for example hypertension, diabetes, and chronic kidney disease), drug therapies with broad potential benefits such as statins, metformin, anti-obesity incretin-based therapies, SGLT2 inhibitors, as well as more holistic LTC reviews in primary care.

#### Minimising impact

For some people with established MLTC, support addressing their needs including consideration of the workload of living with MLTC and the effects on function, quality of life, symptoms, disability, frailty and wellbeing is needed. There is a broad spectrum of need among people with established MLTC and any approach to mitigating the wider impact will need to be graded and proportionate to the level of need—many people with established MLTC will have minimal or no needs, whilst others will have severe symptoms and limitations to their activities of daily living. This requires a multidisciplinary approach often organised at the intersection of health and social care, involving but not limited to care coordinators, doctors, pharmacists, nurses, physiotherapists, occupational therapists and other allied health professional and social care colleagues. This is complemented by chronic disease management programmes, rehabilitation, psychological support, and self-management guidance. A key aspect of minimising impact is the avoidance of health and social care interventions that are no longer of benefit, or do not align with person-centred goals and priorities—quaternary prevention. Deprescribing, avoidance of over-surveillance and over-investigation, de-implementation of interventions that are not adding value for individuals, and adoption of other aspects of minimally disruptive health and social care provision [8], are all important interventions to consider.

There is a need to consider more deeply what frameworks would best reflect the priorities of people living with MLTC in classifying interventions [38], and to ensure frameworks account for harms as well as benefits of treatment. The above categories are based on frameworks designed and used by researchers, clinicians and policymakers; these may not adequately reflect how patients view prevention and treatment of MLTC, and this should be a focus for future work involving patients and the public.

Declaration of competing interests: None.

## MLTC interventions: a life course approach

### Rachel Cooper, Peter Hanlon

Opportunities exist at each stage of life to intervene to prevent, treat and/or mitigate the impacts of MLTC. Use of a life course framework is therefore likely to make the identification of those interventions which are most effective at different life stages more tractable [39] and aid prioritisation. This is not least because it highlights the importance of time (including age, period and birth cohort) and encourages consideration of the accumulation and temporal ordering of different risk factors and long-term conditions across life stages [40–42]; information that is required to inform the identification of the most appropriate intervention strategies at different ages in different population sub-groups.

Figure 1 illustrates some of the key targets for prevention and treatment of MLTC across the life course. This acknowledges that the primary targets will vary across the life course; in earlier life, risk factors for LTC development will need to be prioritised, in mid-life prevention of accumulation of conditions, and in later life treatment and mitigation of symptoms and functional consequences [43] alongside prevention of accumulation of conditions. Understanding the sequence and time course of how MLTC accumulate will be essential to identify not just who to offer interventions to, but when to offer them, and is an area of active research at present. It is also likely that there will be windows of opportunity to intervene at different time horizons across the life course. These windows could include the onset of a new condition, major life events (e.g. pregnancy, retirement), but could also be identified through insights from ageing science, such as recent work suggesting periods of accelerated biological ageing in midlife and early later life [44]. These different areas of focus necessitate different ways of selecting target populations and different types of intervention, but also different sets of outcomes.

Declaration of competing interests: None.

## Types of intervention—proposed taxonomic approaches

### Michelle Collinson, Sally Singh, Stephanie Taylor, Miles Witham

The breadth of interventions that could potentially be deployed to prevent and treat MLTC means that having a framework to describe and group such interventions would be helpful. Such a framework would not only provide a common language for discussion, description and reporting in research and practice but would also facilitate evidence synthesis in this complex field (e.g. in scoping reviews and systematic reviews). We provide two examples to illustrate how a taxonomic approach might be applicable, although developing a full taxonomy is beyond the scope of this paper.

A simple example framework would use broad categories of intervention. These could include pharmacological interventions, exercise and physical behaviour interventions (activity, sedentariness, sleep), nutrition and diet nutrition interventions, device and technology interventions, other behavioural change interventions (e.g. smoking cessation), self-management support and diagnostic and screening interventions. Each intervention could be deployed for prevention or treatment, at different trajectories in the life course to achieve a range of aims. Although we focus in this paper on interventions operating at the level of individuals living with, or at risk of, MLTC, such frameworks could be extended to encompass community, organisational and policy levels of intervention as highlighted in a previous systematic review [45]. The choice of framework for intervention categorisation may need to vary depending on the purpose of classification, and for simple classification schemes as illustrated above, an intervention (or the components thereof) might fit under multiple categories.

A more sophisticated approach could be to build on the World Health Organisation International Classification of Health Interventions (ICHI) taxonomy. This taxonomy uses three domains to classify and code any healthcare intervention [46]: the *target* of the intervention, the *action* by which the intervention exerts its effect and the *means* by which the intervention is delivered. This framework enables a comprehensive description of any intervention at different levels of granularity including for its target population, the ingredients of the intervention, and how those ingredients are delivered. As an example, we might wish to *target* poor mobility as a risk factor for worsening MLTC severity, using the *action* of motivational interviewing, delivered by the *means* of peer support workers. Many of these interventions are likely to be complex interventions [47], composed of multiple interacting components, which presents an additional challenge for classification.

The ICHI approach still has utility for complex interventions; however, for example, we might *target* progression from a single condition to multiple long-term conditions, using the *action* of a comprehensive health assessment and screening process, delivered by the *means* of a multiprofessional primary care team. Such classifications do not remove the need to precisely specify intervention components, particularly for complex interventions. It is also important to note that the taxonomy provides space for interventions that work beyond the level of the individual—examples might include interventions targeting patient/carer dyads, communities (e.g. disability-friendly communities), changes to health care systems (e.g. provision of care coordinators) up to large-scale environmental modifications (e.g. walkable neighbourhoods or longevity-ready cities [48]).

**Fig. 2:**
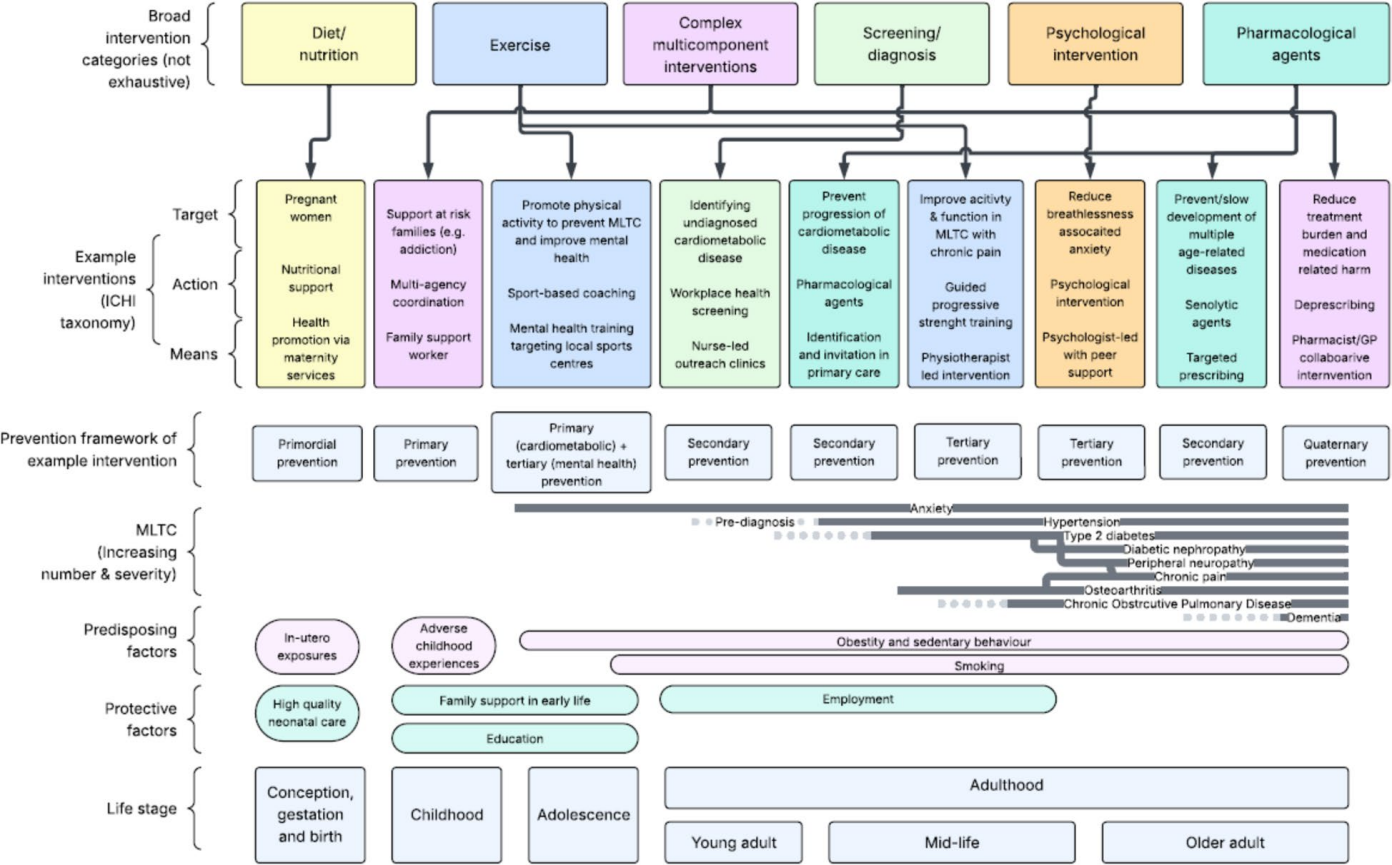
Worked example of how MLTC might develop for an individual across the life course and points for intervention

Figure 2 shows some examples of how a series of different interventions could be characterised by the ICHI taxonomy, and how such interventions might be deployed at different points across the life course as conditions accumulate.

Declaration of competing interests: None.

## Principles of intervention development

### Guru Aithal, Melanie Davies, Sallie Lamb, Claire McDonald, Sally Singh

The heterogeneity of conditions covered by MLTC means that a condition-based approach to developing interventions may not always be optimum. However, it will not always be necessary to develop and deploy interventions that can be used only for MLTC; there are many existing interventions for single conditions that may be modifiable to deliver benefits to a wide range of people living with MLTC. However, there are likely to be a set of interventions which target shared factors or mechanisms underpinning a wide range of conditions, and which will be particularly suited for MLTC by dint of their broad spectrum of benefit across many different conditions. These approaches are not mutually exclusive and can be thought of as existing on a spectrum. Alternative methods of intervention development for broad populations could include:

1. Choice of interventions with pleiotropic effects: Such interventions, by targeting multiple mechanisms of disease, would naturally benefit a wide range of conditions. Exercise interventions are the archetype of this approach [49]. Other pharmacological interventions might include beta blockers (with effects on heart rate in atrial fibrillation, angina symptoms and blood pressure), SGLT-2 inhibitors (via effects on cardiovascular function, renal function and blood glucose) [50] and incretin-based therapies through their weight-reducing and other non-glycaemic pleiotropic mechanisms [51]. Advances in geroscience hold out the possibility of targeting ‘hallmarks of ageing’ [52]—fundamental biological processes that underpin a wide range of different conditions. A promising example here is senolytic agents (that catalyse removal of senescent cells from multiple tissues) which have been proposed as therapies for conditions as diverse as osteoarthritis, cardiovascular disease and dementia [53]. The spectrum of desired pleiotropic effects will vary across the life course and the interventions employed; pleiotropic effects resulting from targeting common risk factors might be important in mid-life, but pleiotropic effects on diverse symptoms might be relatively more important in later life.
2. Addressing common risk factors that underpin multiple conditions: Well-known risk factors for multiple different conditions include cigarette smoking and obesity—both are risk factors amenable to intervention, and there is good evidence for prevention of multiple conditions by addressing these risk factors. Risk factors can be addressed at the individual level, for example via behavioural change (including diet, physical activity and sedentariness) and pharmacological interventions [54] but are also amenable to interventions acting at the level of communities (e.g. walkable neighbourhoods, parkrun) or at the policy level (e.g. clean air legislation, sugar taxes). Whilst some methods to target risk factors will have an impact across the life course, other methods are suitable for targeting risk factors at specific times in life, for example initiatives to prevent childhood obesity and legislation to prevent the uptake of smoking in adolescence. Interventions for risk factors also need to consider whether to target high-risk populations (where cost-effectiveness may be greater) or general populations at lower risk (where the majority of adverse health consequences reside). These approaches are complementary and the characteristics of a specific intervention will determine which target population is appropriate.
3. Addressing issues that matter most to people at risk from, or living with, MLTC: Such a framework could enable the design of interventions that improve the experience of illness (a priority for people living with MLTC) [38] without the increase in multiple forms of ‘work’ (e.g. the work of managing symptoms, finances, appointments or emotional load) that is so often a part of living with MLTC [55]. For people living with MLTC which have symptomatic consequences, a focus on symptom mitigation as the target of interventions provides an important way to improve function and quality of life without this increase in the work of living with MLTC. Examples here might include interventions targeting breathlessness as a symptom [56], as well as interventions to manage chronic pain syndromes [57]. Interventions that focus on systems of care (e.g. provision of care coordinators, integrated outpatient clinics) can also be framed as addressing issues that matter most, as can interventions that focus on minimising treatment burden and the harms of treatment, for example deprescribing interventions and condition specific guidelines that take account of MLTC.

Any interventions to prevent or treat MLTC and their consequences must be flexible enough to be personalised to an individual’s needs and their circumstances and must be deliverable in practice. Many different approaches to intervention development have been described [58], and consensus on the most appropriate way to develop interventions to prevent or treat MLTC and their consequences does not as yet exist. However, ensuring that an appropriate range of stakeholders are integrally involved in the design and evaluation of such interventions is essential [47]. Such involvement needs to be diverse enough to represent the lived experience of a broad range of people at risk of developing MLTC as well as those currently living with MLTC and their carers, as well as stakeholders that are required to ensure effective implementation into practice.

Declaration of competing interests: None.

## Characteristics of effective interventions

### Miles Witham, Andrew Farmer, Nira Shah, Farheen Yameen, Lily Yao

**Fig. 3:**
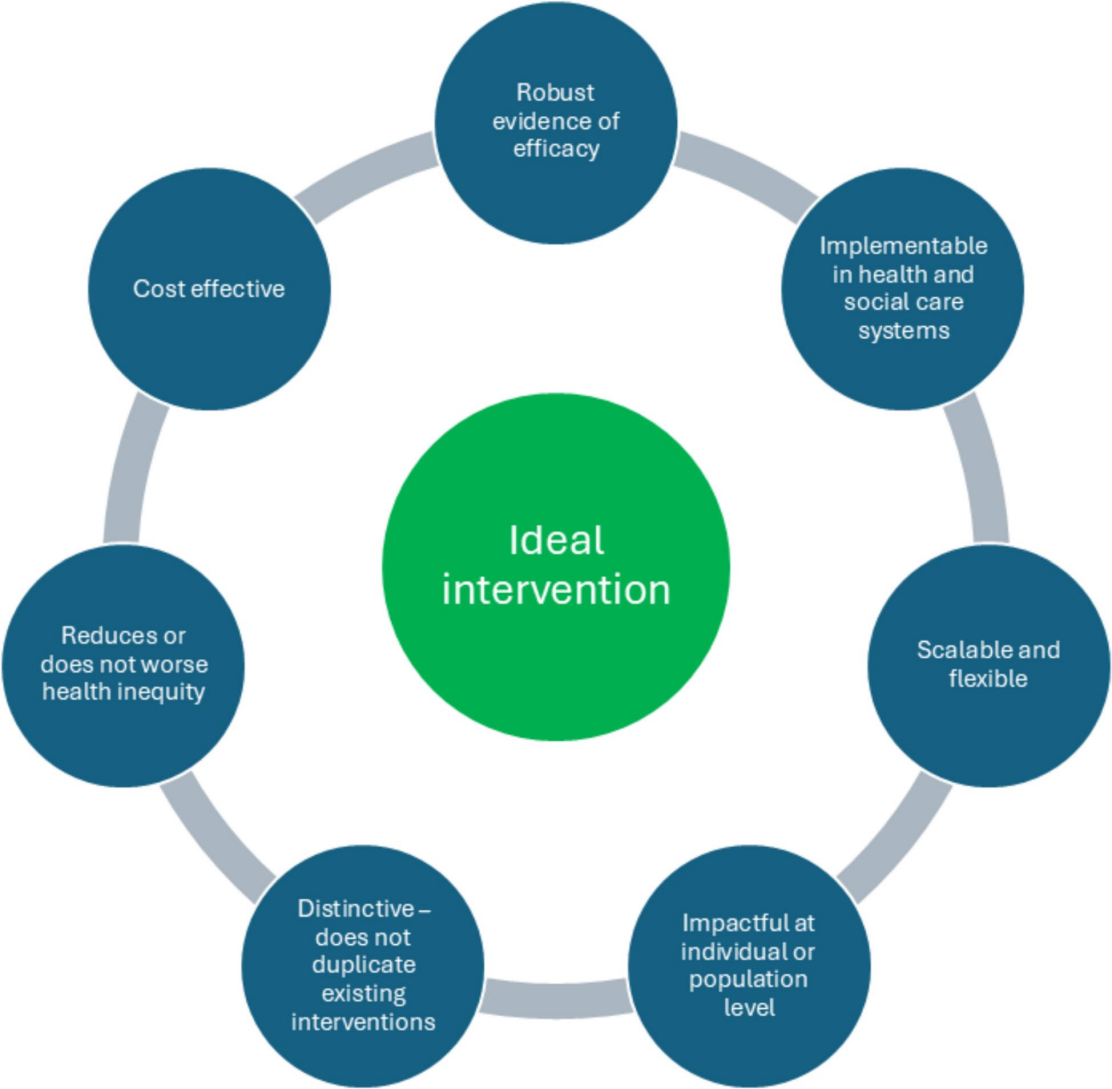
Characteristics of effective interventions

There are many interventions that could potentially be deployed for both prevention and treatment of MLTC, although there is relatively little evidence specific to using interventions in populations living with MLTC. Some interventions are already developed but require effective implementation, some exist but require tailoring to meet the needs of people living with MLTC, and some are novel and require significant research and development before they are fit for purpose with an appropriate evidence base. It is not possible either to research or to implement every potential intervention. Choices therefore need to be made based on which pragmatic interventions are most likely to deliver the biggest impact to the largest number of people in the most cost-effective way. We propose the following points to consider (see Fig. 3), both in deciding whether to deploy an intervention in practice, but also in identifying what gaps in evidence need to be addressed by further development and evaluation:

1. The intervention is underpinned by *robust evidence of efficacy or effectiveness* gathered in the target population. If this is not the case, additional evaluation studies should be conducted. These may be trials (e.g. phase II efficacy trials or phase III effectiveness trials) but could also be evidence from non-trial evaluation studies.
2. The intervention can be *implemented by current health and social care systems* and ideally would be robust to future changes in how health and social care systems are funded and organised. If this evidence does not exist, specific studies may be required to evaluate implementability across a range of health services, populations and condition combinations.
3. Allied to this point, the intervention should be *scalable and flexible*—it can be successfully deployed across different populations and is robust to variations in local health and social care systems to reach a large constituency.
4. The intervention has the potential to deliver either *major improvements in health* and wellbeing to individuals, and/or improvements in health and wellbeing to *large numbers of people* living with, or at risk of, MLTC. Marginal gains to small numbers of people are unlikely to deliver the step change in health outcomes required. This does not preclude the need for interventions that can be tailored to the needs of specific groups; however, such approaches are likely to be essential to successfully engage with a wide range of populations.
5. The intervention *does not duplicate existing or planned interventions* either in content or in intended effect. Novel interventions hold out the possibility of health gains beyond existing approaches. However, this does not preclude repurposing or scaling up of well-established interventions. For either approach, an intervention that seeks to replace, rather than add to, existing interventions is likely to waste resources unless the new approach has clear evidence of superior effectiveness.
6. The intervention *does not worsen (and ideally reduces) health inequity*. This is of particular importance in considering MLTC interventions; factors including socioeconomic position, sex, ethnicity and place are associated with MLTC and inequalities in MLTC across the life course are stark and growing [59–61]. Addressing inequalities and inequities at each level should be a priority for all types of interventions given the disproportionate burden of MLTC in marginalised communities, recognising that many interventions (e.g. behaviour change interventions) risk widening inequalities [62]. Close relationships between research teams and communities are essential to ensure both appropriate co-design of interventions to reduce health inequity and to advocate for delivery of appropriate interventions if they are found to work.
7. The intervention is *cost-effective*, both in absolute terms and when compared to other ‘competitor’ interventions targeted at the same population to prevent or treat MLTC. Although ideally an intervention would save costs compared to standard care, interventions would at least need to meet current thresholds for cost-effectiveness (e.g. UK National Institute for Health and Care Excellence [NICE] [63]), although whether such thresholds adequately capture the complexity of health economic considerations around MLTC is open to debate.

Designing, delivering and evaluating research for people living with MLTC that has widespread impact on practice and health requires a shared language and shared frameworks of understanding. Traditional single-organ disease approaches do not always capture the nuance or complexity required in MLTC research, and thus it is important to ensure that definitions, concepts and taxonomies appropriate to MLTC research are developed and used. We hope that this paper provides an initial step in this direction but we recognise that it should be viewed as a launchpad for further development. Further in-depth review of literature for specific areas of the framework and sourcing the collective wisdom of the MLTC research community (e.g. via consensus exercises) will help to further refine and progress these ideas and concepts in MLTC research.

## Data Availability

No datasets were generated or analysed during the current study.
